# ACUTE KIDNEY INJURY INDUCED BY RHABDOMYOLYSIS IN A YOUNG MALE WITH PREVIOUSLY UNDIAGNOSED HYPOTHYROIDISM

**DOI:** 10.51894/001c.123013

**Published:** 2024-08-30

**Authors:** Sushmita Mantravadi

**Affiliations:** 1 Internal Medicine Garden City Hospital

## 52

### INTRODUCTION

Autoimmune thyroiditis is the predominant cause of primary hypothyroidism, with an incidence of 2.2/100,000/year in males and 498.4/100,000/year in females. Musculoskeletal manifestations range from generalized myalgia and muscle weakness to rare instances of severe rhabdomyolysis leading to acute kidney injury.

### CASE DESCRIPTION

We present a case of a 32-year-old male with no significant medical history, who presented with generalized weakness, myalgia, and edema persisting for three months. The patient engaged in regular-intensity workouts and reported consuming 12 beers several times a week. Physical examination revealed facial edema and proximal muscle weakness. Initial laboratory findings showed elevated creatinine (1.68 mg/dL, baseline 1.0 mg/dL in April 2020), CK (8113 U/L), AST (260 U/L), and ALT (129 U/L). Urinalysis revealed hematuria and proteinuria. Abdominal ultrasound showed diffuse hepatic steatosis with normal kidney appearance. This could not explain the reason for the acute kidney injury and further workup showed TSH (>150 mIU/L) and TPO antibodies (197.9 U/mL). Thyroid ultrasound done subsequently revealed a markedly heterogeneous gland. Treatment included parenteral crystalloids, low-dose prednisone which was empirically added for suspected autoimmune myositis and was later discontinued once the autoimmune panel was negative, and Levothyroxine at the recommended dose. At the time of discharge, his creatinine was reduced to 1.33 mg/dL and CK to 2,298 U/L.

### DISCUSSION

Severe rhabdomyolysis is a rare but significant complication of autoimmune thyroiditis, potentially leading to acute kidney injury (AKI) if not promptly diagnosed and treated. Clinicians often attribute symptoms like myalgia and generalized weakness to hypothyroidism, potentially overlooking severe complications. We recommend routine CPK level monitoring in patients with autoimmune thyroiditis to promptly detect and manage rhabdomyolysis, thereby preventing the progression to AKI.

